# Mitochondrial Complex 1 Inhibition Increases 4-Repeat Isoform Tau by SRSF2 Upregulation

**DOI:** 10.1371/journal.pone.0113070

**Published:** 2014-11-17

**Authors:** Julius Bruch, Hong Xu, Anderson De Andrade, Günter Höglinger

**Affiliations:** 1 Department of Translational Neurodegeneration, German Centre for Neurodegenerative Diseases (DZNE), Munich, Germany; 2 Department of Neurology, Technische Universität München, Munich, Germany; Hospital General Dr. Manuel Gea González, Mexico

## Abstract

Progressive Supranuclear Palsy (PSP) is a neurodegenerative disorder characterised by intracellular aggregation of the microtubule-associated protein tau. The tau protein exists in 6 predominant isoforms. Depending on alternative splicing of exon 10, three of these isoforms have four microtubule-binding repeat domains (4R), whilst the others only have three (3R). In PSP there is an excess of the 4R tau isoforms, which are thought to contribute significantly to the pathological process. The cause of this 4R increase is so far unknown. Several lines of evidence link mitochondrial complex I inhibition to the pathogenesis of PSP. We demonstrate here for the first time that annonacin and MPP^+^, two prototypical mitochondrial complex I inhibitors, increase the 4R isoforms of tau in human neurons. We show that the splicing factor SRSF2 is necessary to increase 4R tau with complex I inhibition. We also found SRSF2, as well as another tau splicing factor, TRA2B, to be increased in brains of PSP patients. Thereby, we provide new evidence that mitochondrial complex I inhibition may contribute as an upstream event to the pathogenesis of PSP and suggest that splicing factors may represent an attractive therapeutic target to intervene in the disease process.

## Introduction

Tauopathies are a heterogeneous group of neurodegenerative diseases with the common feature of intracellular aggregation of the microtubule associated protein tau. They include, but are not limited to, Alzheimer's Disease, Progressive Supranuclear Palsy (PSP), Argyrophilic Grain Disease (AGD), Corticobasal Degeneration (CBD), Pick's Disease and some other forms of frontotemporal dementias. Different tauopathies vary significantly in their clinical and pathological phenotype [1].

In the human central nervous system there are six predominant splicing variants of the *MAPT* gene, encoding tau proteins. These depend on the exclusion or inclusion of exons 2, 3 and 10: 3R0N, 3R1N, 3R2N, 4R0N, 4R1N and 4R2N [2]. 0N signifies the inclusion of neither exon 2 or 3. 1N denotes the inclusion of exon 2 but not 3, whilst 2N denotes the inclusion of both exons 2 and 3. 3R denotes the absence of exon 10, 4R its presence. Exon 10 codes for an additional microtubule binding repeat, so that 4R isoforms have 4 binding repeats, whilst 3R isoforms have only 3.

Across different tauopathies the isoform constitution varies. A common classification of tauopathies, therefore, is between the 3R isoform and the 4R isoform tauopathies [3]. While in healthy adults and in Alzheimer's disease 3R and 4R isoforms are generally in balance, PSP, CBD and AGD feature a relative excess of 4R isoforms [4]. Pick's Disease, conversely, has a relative excess of 3R isoforms. This imbalance is thought to play a major role in the pathogenesis of these tauopathies [5]. 4R isoforms are more prone to aggregation than 3R isoforms [5]. A single mutation in the *MAPT* gene affecting the inclusion of exon 10 to favour generation of 4R tau appears to be sufficient to trigger a tauopathy [6]. This has led to the hypothesis that an excess of 4R tau may be significantly pathogenic. Therefore, reducing the relative amount of 4R may be a strategy for therapy in 4R tauopathies [5], [7].

Alternative splicing of exon 10 is regulated by a combination of *cis*-elements in exon 10 and intron 10, as well as by *trans*-acting factors [2]. It is through these *trans*-acting factors that alternative splicing can be modified and regulated by the cell. They are divided into heterogeneous nuclear ribonucleoproteins (hnRNPs) and serine/arginine-rich (SR) proteins or SR-like proteins. The SR proteins participate in the spliceosome and are thus involved in both constitutive splicing and the regulation of alternative splicing [8]. They are controlled through phosphorylation and acetylation and have been discussed as a potential drug target in the context of cancer treatment [9], [10]. However, so far, the molecular mechanisms leading to preferential generation of 4R tau by alternative splicing of wild-type tau in sporadic 4R tauopathies are not understood.

There are several lines of evidence suggesting a role for dysfunction of the mitochondrial respiratory chain, particularly of mitochondrial complex I, in the pathogenesis of PSP. A study using transmitochondrial cytoplasmic hybrid (cybrid) cell lines expressing mitochondrial genes from persons with PSP found complex I activity to be reduced [11]. Dysfunctional complex I is a major emitter of reactive oxygen species [12] and evidence of oxidative stress has been found in autopsy material of PSP patients [13], [14]. A study using combined phosphorus and proton magnetic resonance spectroscopy has identified evidence for cerebral depletion in high-energy phosphates and increased lactate levels in PSP, a pattern compatible with a primary failure of the mitochondrial respiratory chain [15]. Finally, there is also an epidemiological association between the consumption of soursop fruit containing the mitochondrial complex I inhibitor annonacin [16] and a PSP-like tauopathy on the island of Guadeloupe [17]. Annonacin has been shown to induce a tauopathy *in vitro* in cultured neurons [16], [18], as well as *in vivo* [19]. So far described are four effects of annonacin that are typical features for tauopathies, namely increased tau protein levels, tau hyperphosphorylation, redistribution of tau from the axons to the somatodendritic compartment, and eventual cell death [18]. Here, we explore the effect of complex I inhibition on the alternative splicing of tau.

## Materials and Methods

### Cell Culture

Nunc Nunclon Delta 6-well (for protein and mRNA) or 48-well (for cell assays) plates (Thermo Fisher Scientific, Waltham, MA, USA) were coated with 100 µg/ml poly-L-lysine (Sigma-Aldrich, St. Louis, MO, USA) and 5 µg/ml fibronectin (Sigma-Aldrich). LUHMES (Lund Human Mesencephalic) cells, derived from female human embryonic ventral mesencephalic cells by conditional immortalization [20] (Tet-off v-myc over-expression) were seeded in a concentration of 130,000 cells/cm^2^ to achieve a confluence of 50%. They were then differentiated for 8 days in a medium of DMEM/F12 (Sigma-Aldrich), 1 µg/ml Tetracycline, 2 mg/ml GDNF and 490 µg/ml dbcAMP into post-mitotic neurons [21] with a dopaminergic phenotype [20]. On day 8 post differentiation the cells were treated with 25 nM annonacin, 20 µM 6-OHDA or 10 µM MPP^+^ for 48 h. For the intoxication period the medium was replaced with new medium containing glucose levels reduced to 250 µM, i.e. the physiological concentration in the human brain [22]. For the starving condition, cells were incubated for 24 hours in pure DMEM (Life Technologies, Grand Island, NY, USA) with no additives and no glucose.

### Human Brain Tissue and Ethics Statement

Human fresh frozen brain sections of the *locus coeruleus* area were obtained from The Netherlands Brain Bank, Netherlands Institute for Neuroscience, Amsterdam (www.brainbank.nl). All Material has been collected from donors for or from whom written informed consent for a brain autopsy and the use of the material and clinical information for research purposes had been obtained by The Netherlands Brain Bank in accordance with the Declaration of Helsinki.

### Quantitative Real-Time PCR

RNA from human tissue samples was extracted by grinding the tissue in liquid nitrogen to a powder and then dissolving it in the RA1 buffer supplied as part of the NucleoSpin RNA (Macherey Nagel, Düren, Germany) RNA extraction kit +1% (v/v) 2-Mercaptoethanol (Sigma-Aldrich). RNA from cells was extracted by scraping the cells from the culture plate with RA1 buffer +1% (v/v) 2-Mercaptoethanol. The remaining extraction procedure was according to the manufacturer's instructions for the NucleoSpin RNA kit. RNA concentrations were determined using the NanoDrop 2000c Spectrophotometer (Thermo Fisher Scientific). The RNA was then transcribed into cDNA with the iScript cDNA Synthesis Kit (BioRad, Berkeley, CA, USA) using the manufacturer's instructions. Real-Time PCR was performed on the Applied Biosystems StepOnePlus (Life Technologies) system using TaqMan Universal Master Mix II and TaqMan primers against total *MAPT, MAPT 0N, MAPT 1N, MAPT 2N, MAPT 3R, MAPT 4R, SRSF1, SRSF2, SRSF3, SRSF6, SRSF7, SRSF9, SRSF11* and *TRA2B*. *PSMC1* and *POL2A* were used as reference genes for relative quantification in all tau splicing factor experiments, while *PPIB* and *GAPDH* were used in all tau isoform experiments as they were determined to be the most stably expressed across the respective experimental conditions. All values are relative quantities compared to untreated (control) cells. Three biological repeats with three technical repeats each were analysed. Analysis was conducted with the Applied Biosystems StepOnePlus (Life Technologies) and Qbase+ (Biogazelle, Zwijnaarde, Belgium) software packages. Absolute quantification was performed by creating a standard curve with plasmids containing either the 2N3R or the 2N4R spliced variant of *MAPT* (obtained as a gift from Eva-Maria Mandelkow, DZNE Bonn, Germany). The absolute quantity was computed by deriving the relationship between CT values and absolute quantity with the StepOne Plus software.

### Western Blotting

Protein was extracted from cells using the M-PER Mammalian Protein Extraction Reagent (Thermo Fisher Scientific). The protein solution was frozen at −80°C immediately after retrieval and for a minimum of two hours. The solution was then thawed on ice, vortexed, centrifuged at 5000 g for 15 minutes at 4°C and the supernatant retrieved. Total protein concentrations were determined using the BCA kit (Thermo Fisher Scientific) by heating the samples at 60°C for 30 minutes and measuring the absorption on the NanoDrop 2000c Spectrophotometer (Thermo Fisher Scientific). 20 µg of total protein were then adjusted to equal concentrations between samples by dilution with M-PER and subsequently heated at 95°C for 5 minutes with 1× Roti-Load loading buffer (Carl Roth, Karlsruhe, Germany). SDS-PAGE was performed using precast Gels (anyKD, Bio-Rad) in a tris-glycine running buffer (14.4% glycine, 3% Tris, 1% SDS w/v, Carl Roth). The protein was blotted onto PVDF membrane (Bio-Rad) at 70 V for 65 minutes. The membrane was blocked with 1× Roti-Block solution (Carl Roth) for 1h and then incubated at 4°C overnight under gentle shaking with the primary antibody (table 1) in TBS with 5% BSA (Cell Signaling, Danvers, MA, USA) and 0.05% TWEEN (Sigma-Aldrich). The membranes were then washed and incubated with the appropriate secondary antibody at 1∶2500 (v/v) in 1× Roti-Block solution for 2 h, followed by further washing and exposure to Clarity Western ECL Substrate (Bio-Rad) or, in the case of 4-repeat tau, to **ECL** **solution** (General Electric, Fairfield, CT, USA). Chemiluminescence was detected with the Gel image System (Bio-Rad) and analysed by background subtracted optical density analysis with ImageLab software (Bio-Rad).

**Table 1:** Primary Antibody Concentrations Used.

Antigen	Clone	Species	Concentration (v/v)	Company
Human tau	HT7	Mouse	1:1000	Pierce Antibodies, Thermo
3-repeat tau	8E6/C11	Mouse	1:500	Millipore
4-repeat tau	1E1/A6	Mouse	1:300	Millipore
Actin (I-19)	Polyclonal	Goat	1:2500	Santa Cruz Biotechnologies

### siRNA Silencing

LUHMES cells were seeded out and differentiated as described above and allowed to adhere to the plate floor for 4 h. siRNA (Sigma-Aldrich) targeted against SRSF2 (final concentration 200 nM) and Lipofectamine RNAiMAX (Life Technologies) (final concentration 1.2 µl/ml) were dissolved in separate aliquots of medium (OptiMEM, Life Technologies). The diluted siRNA was then added to the diluted Lipofectamine RNAiMAX. The combined solution was then allowed to incubate for 20 minutes before being added to the cells.

### ATP Assay

ATP assays were conducted using the ATP test kit by Lonza according to the manufacturer's instructions. Luminescence was read with the FLUOstar Omega platereader (BMG Labtech). The data was analysed using the MARS Data Analysis Software (BMG Labtech).

### MTT Assay

Thiazolyl Blue Tetrazolium Blue (MTT) (Sigma Aldrich) was dissolved in sterile PBS to a concentration of 5 mg/ml. This stock solution was added to the cells in culture medium to achieve a final concentration of 0.5 mg/ml. The 48-well culture plate was then incubated at 37°C for 1 h, the medium removed completely and frozen at −80°C for 1 h. The plate was then thawed, 300 µl DMSO (AppliChem, Darmstadt, Germany) was added per well and the plate was shaken to ensure complete dissolution of the violet crystals. 100 µl from each well were transferred to a new 96-well plate and the absorbance was read with the platereader at a wavelength of 590 nm (reference wave length 630 nm). The data was analysed using the MARS Data Analysis Software (BMG Labtech).

### Statistics

Prism 6 (GraphPad Software, La Jolla, CA, USA) was used for statistical calculations and for the creation of line and bar graphs. Results were compared by 2-way ANOVA with Sidak post-hoc test, unless stated otherwise. Data are shown as mean ± SEM. P<0.05 was considered significant.

## Results

### Annonacin Causes an Upregulation of the Tau 4-Repeat Isoform

We first characterized expression of tau isoforms in LUHMES cells, a cell culture line of human mesencephalic neurons, derived from female human embryonic ventral mesencephalic cells by conditional immortalization (Tet-off v-myc over-expression) [21]. These cells start expressing the 4-repeat (4R) isoform of tau from day 8 post differentiation into a neuronal phenotype. On day 10, 4R-spliced mRNA makes up 3.9% ±0.3 (n = 3) of the total MAPT mRNA. We used this human neuronal model for the present work as rodent cells express only 4R tau.

**Figure 1:**
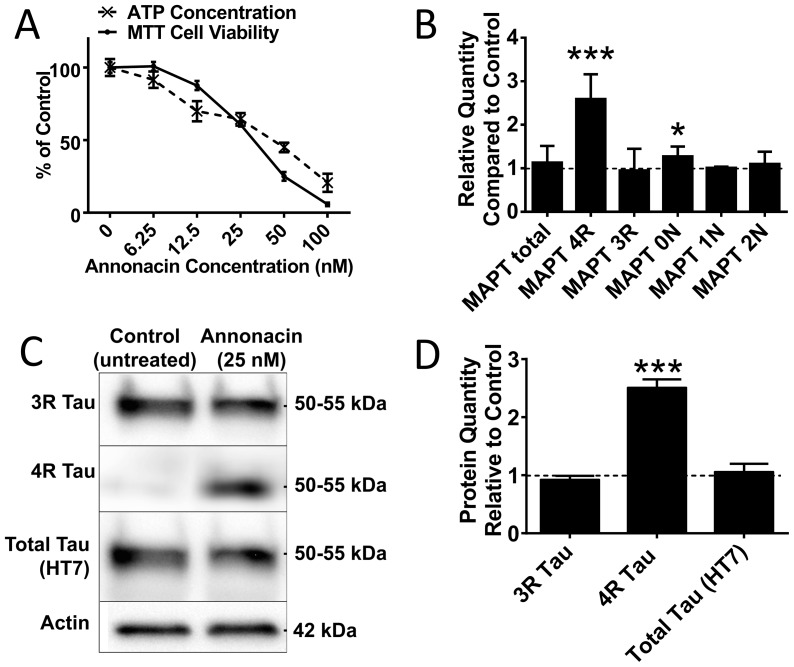
Annonacin Causes an Upregulation of the 4R Isoforms of Tau. A) LUHMES neurons were treated with different concentrations of annonacin for 48 h from day 8–10 post differentiation (n = 12). The MTT test, a measure for mitochondrial reducing function, and ATP concentration, are expressed as a relative percentage compared to untreated control cells. B) 4R isoform (exon 10) mRNA is upregulated with annonacin treatment. Quantitative PCR results showing the relative quantity of mRNA for different *MAPT* splicing variants in cells treated with 25 nM annonacin for 48 h from day 8–10 post differentiation compared to untreated cells (dotted line). 3 biological repeats with 3 technical repeats each. ***: p<0.001, *: p<0.05 vs. untreated cells (2-way ANOVA with Sidak's post-hoc test). C) 4R isoform protein is upregulated with annonacin treatment. Western blot for 3R and 4R isoforms of tau protein, as well as total tau (detected with the HT7 antibody). LUHMES cells were either left untreated or treated with 25 nM annonacin. Actin was used as loading control. D) Quantification of figure 1C. Results show the relative quantity (fold-change) compared to untreated control cells (relative quantity  = 1, represented by dotted line). 3 biological repeats. ***: p<0.001 vs. untreated cells (2-way ANOVA with Sidak's post-hoc test).

When treated with annonacin at a concentration of 25 nM for 48 h from days 8 to 10 post differentiation, LUHMES cells remain 60.7±0.4% viable (MTT assay) with an ATP concentration of 64±1% compared to that of untreated cells (figure 1A).

Under these conditions we observed the mRNA of the 4R isoforms of tau to be upregulated significantly (figure 1B) compared with untreated cells, as determined by quantitative PCR. There was no significant change in the relative quantity of 3R isoforms. The level of inclusion of exons 2 and 3 also did not change significantly, although there was a slight increase in the amount of 0N isoforms. This indicates that annonacin selectively increases inclusion of exon 10 with no or little relative effect on the alternative splicing of the other exons.

We also observed an upregulation of the 4R tau isoforms on the protein level by Western blot (figure 1C). 3R tau was again not significantly changed. The level of upregulation at the protein level is very similar to that on the mRNA level, suggesting a tight correlation between the regulation of alternative splicing and the isoform distribution seen at the protein level. There was no significant increase in the amount of total tau with annonacin, probably due to the greater proportion of 3R isoforms in LUHMES cells of this age.

### The Splicing Factor SRSF2 Is Necessary for Annonacin-Mediated Alternative Splicing

We next explored the mechanism of how annonacin induces this isoform change. We tested 10 splicing factors known to influence the inclusion or exclusion of exon 10 in the MAPT gene [2] by quantitative PCR. An overview of the splicing factors tested is shown in table 2.

**Table 2:** Overview of the splicing factors known to influence MAPT exon 10 alternative splicing.

Splicing factor	Target *cis*-element	Effect on exon 10 splicing
SRSF1 (SRp30a, ASF)	PPE	Inclusion
SRSF2 (SRp30b, SC35)	SC35-like	Inclusion
SRSF3 (SRp20)	ND	Exclusion
SRSF4 (SRp75)	ND	Exclusion
SRSF6 (SRp55)	ND	Exclusion
SRSF7 (9G8)	ISS	Exclusion
SRSF9 (SRp30c)	ND	Inclusion
SRSF11 (SRp54)	PPE	Exclusion
TRA2B	PPE	Inclusion

Source: Adapted from [2].

**Figure 2:**
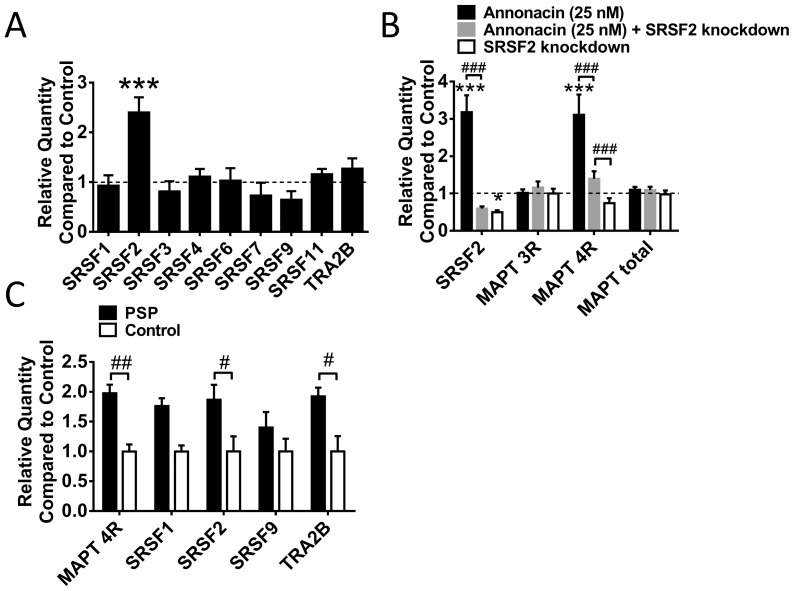
SRSF2 is a Critical Player in Annonacin Mediated Tau Alternative Splicing. A) Quantitative PCR results for 9 different splicing factors known to have an effect on exon 10 alternative splicing (table 2). Data shown are relative quantities compared to untreated cells (dotted line). Only SRSF2 was elevated significantly with annonacin treatment. All other splicing factors tested were not significantly elevated. 3 biological repeats with 3 technical repeats each. ***: p<0.001, *: p<0.05 vs. untreated control (2-way ANOVA with Sidak's post-hoc test). B) Quantitative PCR results for LUHMES cells on day 10 post differentiation treated with SRSF2 knockdown siRNA for 10 days and/or with annonacin for 48 h. 3 biological repeats with 3 technical repeats each. ***: p<0.001 vs. untreated control (dotted line); ###: p<0.001 (2-way ANOVA with Sidak's post-hoc test). C) Quantitative PCR results for the 4 splicing factors known to increase MAPT exon 10 inclusion in *locus coeruleus* tissue of four PSP patients and five controls without neurodegenerative diseases. 3 biological repeats with 3 technical repeats each. #: p<0.05, ##: p<0.01 (2-way ANOVA with Sidak's post-hoc test).

We found SRSF2 to be the only splicing factor significantly upregulated with annonacin treatment that is known to promote the inclusion of exon 10 (figure 2A). This prompted us to explore whether SRSF2 has a functional role to play in annonacin mediated 4R upregulation. We knocked down *SRSF2* with siRNA starting from 6 hours post differentiation in LUHMES cells and treated these cells with annonacin from days 8–10, as in the previous experiments. At day 10, *SRSF2* was reduced by half. In spite of this incomplete silencing efficiency, the 4R isoform of *MAPT* in annonacin treated cells was reduced dramatically compared to untreated cells (figure 2B). This suggests that SRSF2 plays a critical role in the upregulation of the 4R MAPT isoforms seen upon annonacin treatment.

### More Splicing Factors Are Upregulated in Human PSP

We also tested splicing factor expression levels in human brain tissue of the *locus coeruleus* of 4 PSP patients and five control patients free of psychiatric or neurodegenerative diseases (table 3). This time, however, we limited our analysis to those splicing factors known to increase *MAPT* exon 10 inclusion. We confirmed the increase of the 4R isoform in the PSP patients compared to the controls (figure 2C). Expression of the splicing factors *SRSF2* and *TRA2B* was also increased significantly. This suggests that the increase in 4R isoforms seen with annonacin treatment may account partly for the mechanism by which 4R isoform tau is upregulated in PSP.

**Table 3:** Overview of Human Tissue Used.

Case Number	Diagnosis	Cause of Death	Age at death	Braak Stage	Sex	Postmortem delay (hours: minutes)
P1	PSP	“Natural death”	73	2C	Male	4:20
P2	PSP	Acute heart failure	70	3	Male	6:50
P3	PSP	Aspiration pneumonia	73	2	Male	6:15
P4	PSP	Urinary tract infection	70	1A	Male	5:20
C1	Non-demented control	Pancreas carcinoma	70	0	Male	7:30
C2	Non-demented control	Prostate cancer	69	0	Male	5:55
C3	Non-demented control	Lung emboli (clinical suspicion)	73	0	Male	24:45
C4	Non-demented control	Sepsis	71	1	Male	7:40
C5	Non-demented control	Myocardial infarction	67	1B	Male	18:35

### 4R Tau Upregulation Occurs with Other Complex I Inhibitors but Not Oxidative Stress

**Figure 3:**
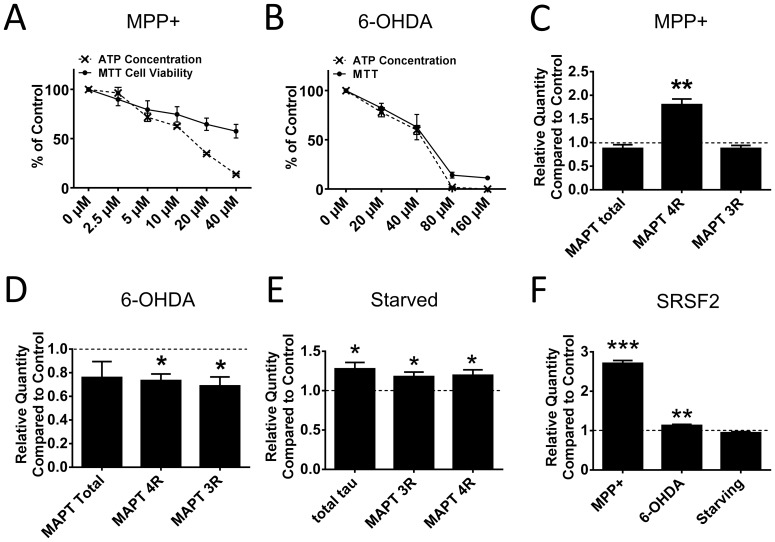
The 4R Isoform Shift Can Be Reproduced with Another Complex I Inhibitor but not with the Oxidative Stressor 6-OHDA. A) ATP concentration and MTT cell viability in LUHMES cells as measured by the MTT assay for different concentrations of 6-OHDA. Treatment was for 48 h from day 8–10 post differentiation. n = 12. B) ATP concentration and MTT cell viability in LUHMES cells as measured by the MTT assay for different concentrations of MPP+. Treatment was for 48 h from day 8–10 post differentiation. n = 12. C) Quantitative PCR results of MAPT splicing variants for LUHMES cells treated with 10 µM MPP^+^ for 48 h from day 8–10 post differentiation. 3 biological repeats with 3 technical repeats each. **: p<0.01 vs. untreated cells (dotted line), (2-way ANOVA with Sidak's post-hoc test). D) Quantitative PCR results of MAPT splicing variants for LUHMES cells treated with 20 µM 6-OHDA for 48 h from day 8–10 post differentiation. 3 biological repeats with 3 technical repeats each. *: p<0.05 vs. untreated cells (dotted line), (2-way ANOVA with Sidak's post-hoc test). E) Quantitative PCR results of MAPT splicing variants for LUHMES cells starved of nutrients and glucose for 24 h from day 8–9 post differentiation. 3 biological repeats with 3 technical repeats each. *: p<0.05 vs. untreated cells (dotted line), (2-way ANOVA with Sidak's post-hoc test). F) Quantitative PCR results of SRSF2 for LUHMES cells treated with 10 µM MPP^+^ or 20 µM 6-OHDA for 48 h from day 8–10 post differentiation or starved for 24 h from day 8–9 post differentiation. 3 biological repeats with 3 technical repeats each. *: p<0.05, **: p<0.01 vs. untreated cells (dotted line), (2-way ANOVA with Sidak's post-hoc test).

We tested whether 4R isoform upregulation upon annonacin treatment is a non-specific consequence of neuronal injury, specific to mitochondrial complex I inhibition or even more specific to annonacin. We therefore repeated the experiment with 1-methyl-4-phenylpyridinium (MPP^+^), another complex I inhibitor, 6-hydroxydopamine (6-OHDA), a neurotoxin known to be neurotoxic primarily through oxidative stress [23] and by starving the cells of glucose and nutrients. As shown in figures 3 A and B, a comparable level of toxicity and ATP reduction to that of 25 nM annonacin is achieved by 6-OHDA at a concentration of 20 µM (22% ATP reduction) and by MPP^+^ at a concentration of 10 µM (37% ATP reduction). Therefore, we decided to use these concentrations to test the MAPT isoform changes with these toxins.

**Figure 4:**
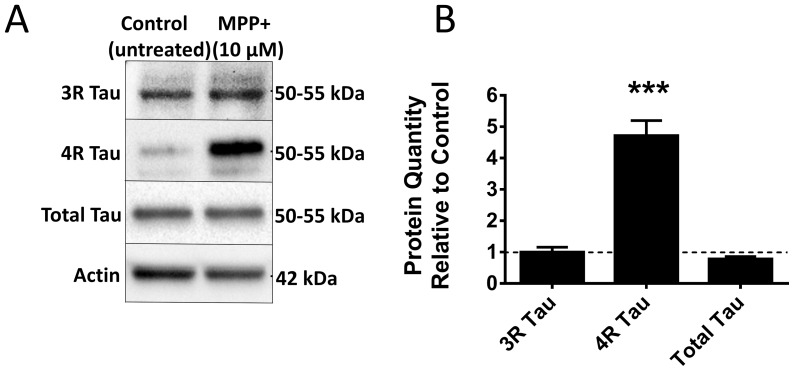
The 4R Isoform was upregulated in the protein level with MPP^+^ treatment. A) 4R isoform protein is upregulated with MPP^+^ treatment. Western blot for 3R and 4R isoforms of tau protein, as well as total tau (detected with the HT7 antibody). LUHMES cells were either left untreated or treated with 10 µM MPP^+^. Actin was used as loading control. B) Quantification of figure 1G. Results show the relative quantity compared to the untreated control cells (dotted line). 3 biological repeats. ***: p<0.001 vs. untreated cells (2-way ANOVA with Sidak's post-hoc test).

With MPP^+^ treatment we observed a significant increase in exon 10 inclusion on the mRNA level by qPCR (figure 3C) and in the levels of 4R tau isoforms by Western blot (figure 4A, B) compared to controls, as with annonacin. With 6-OHDA treatment and with starvation we only observed a slight reduction in both 4R and 3R isoforms. In all three cases the inclusion of exons 2 and 3 did not increase (data not shown). This would suggest that complex I inhibition in general and not oxidative stress or neuronal suffering per se is responsible for the increased level of exon 10 inclusion observed with annonacin.

Finally, we explored the role of *SRSF2* in these observations. We found that MPP^+^ also acts via SRSF2 upregulation and that there is no *SRSF2* upregulation with 6-OHDA treatment or starvation.

## Discussion

### Mitochondrial Complex I Inhibition Reproduces the 4R Isoform Shift Seen in Several Tauopathies

In this paper we have been able to add the increase in 4R tau isoforms as an additional feature to the list of characteristics of PSP that annonacin treatment reproduces in cell culture. This makes annonacin treated neurons a good model for PSP and potentially other sporadic 4R tauopathies. It is unique in the fact that it does not rely on any genetic modification of the *MAPT* gene or artificial overexpression. The fact that it reliably produces an increase in the 4R tau isoforms would also allow it to be used to screen, test and develop candidate drugs targeting tau alternative splicing – something that would not be possible with overexpression-based models of tauopathy.

However, the effect on alternative splicing is not specific to annonacin. Rather, it seems to be related to mitochondrial complex I inhibition more generally. This is suggested by the fact that we have observed the same increase in 4R tau isoforms with MPP^+^, another complex I inhibitor. In fact, other features of tauopathy have also been reproduced by other complex I inhibitors [24], [25]. However, due to the epidemiological evidence from Guadeloupe strongly linking annonacin consumption to a PSP-like tauopathy, annonacin makes a particularly convincing case as a cell culture based model for PSP. The only drawback of this model relying on immature human neurons is that despite the upregulation of 4R tau, after 10 days there still appears to be overall more 3R than 4R tau, whereas in adult human brain neurons, 3R and 4R are more or less balanced.

However, it is not yet fully understood to what extent the relative increase in the 4R tau isoform contributes to neurotoxicity or impairment of neural functioning. 4R tau isoform increases are only seen in a selection of tauopathies and are region specific. In Alzheimer's disease, there is no abnormal upregulation of 4R isoforms. In PSP, there is some evidence that the 4R isoform may not be upregulated in the frontal cortex, despite the existence of tau pathology in this region [26]. On the other hand, patients with FTDP-17 due to mutations that exclusively affect tau alternative splicing and result in an increase of 4R tau, are evidence that an upregulation of the 4R isoforms is sufficient to start tau aggregation [2], [6].

### SRSF2 Forms the Link Between Complex I Inhibitors and the Increase in 4R Isoforms

We have identified SRSF2 as a mediator essential for mitochondrial complex I inhibitor induced exon 10 inclusion. The fact that a knockdown of SRSF2 reverses the annonacin induced increase in 4R tau confirms that SRSF2 plays a necessary role for this isoform shift.

SRSF2 is controlled by several kinases including SRPK, AKT, topoisomerase I and CLK/STY family kinases, as well as lysine acetylation [27]. The histone deacetylase inhibitor sodium butyrate has already been demonstrated to increase SRSF2 levels [28], whilst the kinase activity of topoisomerase I can be inhibited with the antitumour drug NB-506 [9]. This suggests that, at least indirectly, SRSF2 is a potentially drugable target.

In our annonacin-treated cell cultures, which might be considered to be an acute model of a sporadic tauopathy, inhibition of SRS2 prevented the 4R isoform shift of tau but not the cell death induced by annonacin. This suggests that, in this model, the 4R tau is not necessary for cell death, since neurons might rather die from reduced energy production [18]. This does, however, not exclude that in a more chronic situation with even higher levels of 4R tau this isoform shift may become the predominant cause of neuronal dysfunction and death.

### Complex I Inhibition Is Unlikely to Explain All of the Increase in 4R Isoforms in PSP

In human PSP patients both the SRSF2 and TRA2B splicing factors are upregulated. This suggests that the 4R upregulation is not exclusively due to complex I inhibition, as in that case we would have expected only SRSF2 to be upregulated. Therefore, exploring upstream events leading to TRA2B upregulation may lead to insights on further reasons for the increase in 4R tau isoforms in some tauopathies. It would also be interesting to compare the splicing factor expression levels in 3R tauopathies versus 4R tauopathies.

If SRSF2 is confirmed to be a key player in mediating the 4R isoform upregulation in PSP and other 4R tauopathies, this would make it a suitable drug target for reducing this isoform shift.

## Conclusion

In summary, we can conclude that SRSF2 is a necessary mediator for mitochondrial complex I inhibitor induced tau 4R isoform upregulation. As SRSF2 is also increased in PSP patients this suggests mitochondrial complex I inhibition may play at least a partial role in the pathogenesis of 4R tauopathies such as PSP. However, other mechanisms are also likely to contribute.
